# Takayasu arteritis with pyoderma gangrenosum: case reports and literature review

**DOI:** 10.1186/s41927-019-0098-z

**Published:** 2019-11-05

**Authors:** Xuehan Zhang, Yang Jiao

**Affiliations:** 1 Department of Health Care, Peking Union Medical College Hospital, Chinese Academy of Medical Sciences & Peking Union Medical College, Beijing, China; 2 Department of General Internal Medicine, Peking Union Medical College Hospital, Chinese Academy of Medical Sciences & Peking Union Medical College, No. 1, Shuaifuyuan, Wangfujing St., Beijing, 100730 China

**Keywords:** Takayasu arteritis, Pyoderma gangrenosum, Skin lesions

## Abstract

**Background:**

Takayasu arteritis is a rare, chronic inflammatory arteriopathy affecting mainly the aorta and its branches. Many skin manifestations have been reported in association with this disease. Pyoderma gangrenosum is a skin complication that is a neutrophilic dermatosis characterized by destructive, necrotizing and noninfective skin infiltration. However, there are no related records on these conditions in Chinese patients.

**Case presentation:**

We reported two Chinese female patients presenting with pyoderma gangrenosum associated with Takayasu arteritis. Pyoderma gangrenosum preceded Takayasu arteritis in both patients. Their skin lesions were diagnosed as pyoderma gangrenosum through skin biopsy and relieved after treating with steroids and immunosuppressants. During the follow-up, both patients developed symptoms caused by vascular stenosis and occlusion, such as dizziness and weakness of upper limb. The results of aortic angiography revealed multiple large arteries narrowed and blocked. According to the criteria of the American College of Rheumatology, the vasculitis in both patients were classified as Takayasu arteritis. Since there was scant evidence of active inflammation and the skin lesions were stable, neither of them was given strong immnosuppressive therapy. The PubMed database was also searched and 16 related well-documented cases of Takayasu with pyoderma gangrenosum were reviewed and summarized.

**Conclusions:**

Pyoderma gangrenosum could occur at any stage of the Takayasu arteritis disease process. No correlation was found between the location of the skin lesions and the clinical severity and scope of Takayasu arteritis. It is important to remember the rare possibility of Takayasu arteritis in patients with skin lesions indicative of pyoderma gangrenosum of unknown aetiology. Obtaining the relevant history and regular monitoring of the arteries are necessary.

## Background

Takayasu arteritis (TA) is an uncommon, chronic granulomatous large vessel vasculitis that predominantly affects the aorta, its main branches and the pulmonary arteries [1]. This disease is more prevalent in Asian countries, though it is reported worldwide. TA occurs most frequently in patients who are in their second and third decades and predominantly in females [2]. The disease typically presents with malaise, fever, weight loss and symptoms due to arterial occlusion. This initial “prepulseless” stage may overlap or be followed by a second “pulseless” stage characterized by inflammation of the media and adventitial layers of large vessel walls, resulting in vascular stenosis and occlusion [3].

Cutaneous manifestations of TA are uncommon, only being observed in up to 2.8–28% of patients [4]. They include pyoderma gangrenosum (PG), erythema nodosum and erythema induratum. Pathology features of PG are necrotizing, destructive and non-infectious ulceration characterized by neutrophil infiltration of the skin [5]. Most cases of PG associated with TA have been observed in Japan, with no cases reported in Chinese patients.

We report two cases of young female patients who presented with PG that widely affected the extremities and face and were subsequently diagnosed with TA due to severe large artery involvement after a long follow-up period. A review of the literature identified similar cases of TA with PG.

## Case presentation

### Case 1

This patient was a 28-year-old Chinese woman who was diagnosed with TA 4 years after skin manifestation. When she was 24 years old in 1996, she presented with fever, as well as multiple pustules and subcutaneous abscesses within an erythaematous plaque in the medial part of the bilateral thighs and buttocks. She did not respond to treatment with several antibiotics. Her complete blood count showed mild leucocytosis of 13.0 × 10^9^/L (3.5–9.5 × 10^9^/L), with 82% neutrophils, haemoglobin of 9.5 g/dL (11.5–15.5 g/dL), and a platelet count of 310 × 10^9^/L (100–350 × 10^9^/L). C-reactive protein (CRP) was 20.2 mg/L (0-8 mg/L), and the erythrocyte sedimentation rate (ESR) was 105 mm/1st hour (< 20 mm1st hour). Renal and liver functions, urinalysis, and coagulation profile were in normal range. A skin biopsy of the upper extremity revealed neutrophil infiltration of the upper layer of the epidermis and dermis, forming abscesses, which led to the diagnosis of PG. Multiple bacterial cultures from pustules and abscesses showed no evidence of bacterial infection. Subsequently, the patient was treated with oral prednisolone (40 mg/d), cyclosporine (100 mg/d) and triptolide (60 mg/d). With remission of the disease, the prednisolone dose was gradually tapered and halted after half a year. In June 1998, she presented with rash with ulcerated pustules on the surface in the left lower extremity. The rash was gradually relieved after receiving asaisone (8 mg/d). Glucocorticoid was stopped in February 2000.

In April 2000, 3 months before admission, she developed dizziness and had a fainting episode. She was pulseless in both upper limbs. Physical examination upon admission to our hospital showed multiple scar-like lesions on the skin of her extremities, with surface shrinkage. Neither of her radial and brachial arteries were palpable. Blood pressure of both upper extremities was 0 mmHg. Lower limb pulses were normal, and blood pressure was 160/70 mmHg on the right side and 168/80 mmHg on the left. The results of aortic angiography revealed that the right innominate artery (subclavian artery, common carotid artery) was completely obliterated. In addition, collateral circulation had formed. The left common carotid artery was entirely occluded. The initial segment of the left vertebral artery had stenosis, and the left vertebral artery was enlarged. No obvious abnormalities were found in the right upper extremities. The pathological results of a skin biopsy revealed mild oedema, infiltration of inflammatory cells and hyperplasia of fibrous tissue in the lower layer of the epidermis. However, the medium blood vessels in the subcutaneous tissue were normal. The ESR was 14 mm/h, and the CRP level was 0.36 mg/L, which were both in the normal range. The results of the following tests were normal: liver function, blood urea nitrogen, creatinine and electrolytes. According to the criteria of the American College of Rheumatology (ACR), the vasculitis in this woman was classified as TA [6]. Because there was no sign of active TA and the skin lesion was stable, she was prescribed aspirin (75 mg/d) and ticlopidine (250 mg/d). The patient’s condition has remained stable during follow-up.

### Case 2

In 2010, a 17-year-old Chinese female developed mottling rashes in her right thigh that expanded to both sides. The initial inflammatory lesion subsequently expanded peripherally and degenerated centrally, leading to ulcer and pustule formation. Prednisone and cyclosporin A relieved the skin lesion, and the medicine was then tapered. Three years later, she developed multiple pustules in her left upper extremity. Investigations revealed white cell count of 20.53 × 10^9^/L (3.5–9.5 × 10^9^/L), with 88.5% neutrophils and ESR of 120 mm/1st hour (< 20 mm/1st hour). A skin biopsy of the upper extremity was performed at the local hospital. The results showed a large number of neutrophils, plasma cells, lymphocytes and multinucleated giant cells infiltrating the dermis and subcutaneous tissue. No bacteria were found in the subcutaneous tissue. PG was diagnosed, and she was prescribed methylprednisolone with a gradually decreasing dose. She experienced 3 PG relapses during the next 4 years, and the skin lesion gradually affected her left buccal region and right extremity. After treatment with methylprednisolone, there were only a small number of rashes on her upper left arm remained and no more massive pustules were present on her extremities.

**Fig. 1:**
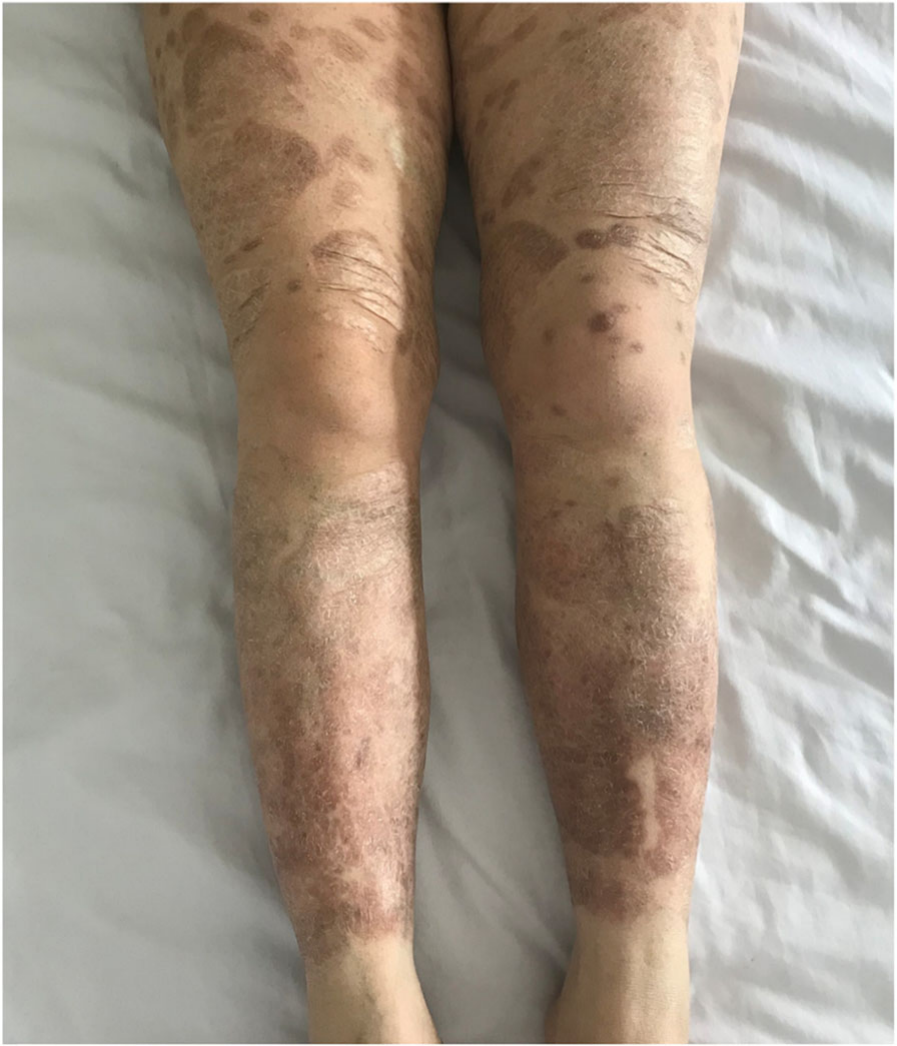
Diffuse old scars of pyoderma gangrenosum on the lower extremities

In June 2017, 6 months before admission to our hospital, the patient presented with left arm weakness followed by dizziness. The clinical manifestations gradually increased, and thus she visited our institution. At admission, there were massive pigmentation and scars on her extremities and face (Fig. 1) but no ulcers remained. She was also in good general condition. Physical examination revealed no pulse in her left cervical, left radial and right dorsalis pedis arteries. Blood pressure was 93/76 mmHg in the left upper arm and 106/46 mmHg in the right arm; blood pressure in the left and right lower limbs were 158/51 mmHg and 126/55 mmHg, respectively. A spray-like noise was heard from the left subclavian artery and bilateral femoral artery. Laboratory investigations showed that her ESR had increased to 43 mm/1st hour (< 20 mm/1^st^ hour), and the CRP level increased to 20.55 mg/L (0-8 mg/L). White and red blood cell counts, renal and liver function tests, serum complements, rheumatoid factor, antiphospholipid antibodies, antinuclear antibody, and antineutrophilic cytoplasmic antibodies were within normal range or negative.

Colour Doppler ultrasonography showed that the left carotid artery was narrowed and blocked, the artery wall was thickened, structure was unclear in the left subclavian artery, and the bilateral femoral arteries, superficial femoral arteries and the left popliteal artery were thickened. Subsequent computed tomography angiography showed that the ascending aorta, the aortic arch and its branches, the descending aorta, and the abdominal aorta (mainly at the L3 vertebral body level) and its branches were thickened and irregular. The celiac stem and mesenteric artery were occluded, with multiple collateral circulation formation. The main hepatic artery and splenic artery exbihited severe stenosis in the lumen. According to the ACR criteria, this woman was diagnosed with TA [6]. In view of scant evidence of active inflammation, and the development of the disease to the stenotic stage, the patient was given methylprednisolone (4 mg/d), mycophenolate (100 mg/d) and aspirin (100 mg/d). The patient’s condition remains stable during regular follow-up.

## Discussion and conclusions

PG in association with TA is rare. To date, 1435 hospitalized patients have been diagnosed with TA at Peking Union Medical College Hospital, and only two were diagnosed with PG. We searched for previous English-language literature and found 16 related well-documented cases of TA with PG [2–4, 7–18] (Table 1). With our 2 cases, a total of 18 cases were reviewed.

**Table 1:** Review of cases of Takayasu arteritis with pyoderma gangrenosum in the literature

Authors, country, year	Sex	Racial or ethnic group	Location of PG	Age at onset of the first manifestations, years			Therapy	Systemic symptoms	First vascular manifestation	Vascular involvement	Other complications
				Systemic	Cutaneous	Vascular					
Okamura et al. [6] Japan, 2017	M	Japanese	First in left buccal region and then spread to cheek, trunk and lower legs	33	37	33	Betamethasone 1 g/d and tacrolimus 3 mg/d for TA, prednisolone 50 mg/d for PG	Fever	Left cervical pain followed by dizziness		
Vettiyil et al. [7], India, 2017	F	Indian	Dorsum of left foot	8	10	8	Prednisolone (1 mg/kg/d) and mycophenolate mofetil for TA and PG		Pain andclaudication pain over legs and back with breathlessness on exertion	Dilation of the ascending aorta, the left common carotid artery, left subclavian, right subclavian and axillary were narrowed at the origin and proximal portion	Chronic recurrent multifocal osteomyelitis
Loetscher et al. [3], Switzerland, 2016	F	Caucasian	Lower extremities	No record	42	43	Prednisone 50 mg/d, cyclosporine, methotrexate			Proximal occlusion of the left subclavian artery, wall thickening of the aortic arch and the subclavian artery	Erythema nodosum
Barrera-Vargas et al. [2], Mexico, 2015	F	Mexican	First in lower limbs and then to head, neck, upper and lower extremities	20	20	26	Prednisone 1.5 mg/kg/d and thalidomide 200 mg/d for PG, three methylprednisolone pulses (1 g/d) and prednisone 1 mg/kg/d and methotrexate 15 mg/week for TA	Fever	Sudden amaurosis in left eye	Both the carotid and vertebral system, all the supra-aortic branches, right pulmonary artery	CT revealed a cystic lesion with an air-fluid level in the lower right pulmonary lobe
Barrera-Vargas et al. [2], Mexican, 2015	M	Mexican	Neck, trunk, upper and lower extremities	25	17	26	Prednisone and cyclosporine, and later thalidomide 200 mg/d, dapsone 100 mg/d, azathioprine 100 mg/d for PG. Prednisone 1 mg/kg/d, thalidomide 200 mg/d, dapsone 100 mg/d, azathioprine 100 mg/d for TA	No	Light-headedness and syncope	Aortic dilatation, narrowing thickening of the wall of the left carotid artery, and celiac trunk stenosis, as well as thickening at the origin of the superior mesenteric artery and left renal artery	Pulmonary nodules,histopathologic analysis showing granulomatous chronic inflammation with giant cells
Futaki et al. [8], Japan, 2009	F	Japanese	Neck, face and forearm	28	28	29	Prednisolone 30 mg/d	Intermittent fever, sore throat and cough		Pulmonary artery	TA without typical symptoms
Minagawa et al. [9], Japan, 2009	F	Japanese	Left lower leg	21	16	21	Tacrolimus for PG, prednisolone 25 mg/d for TA and necrotizing vasculitis	Episodic general fatigue and low-grade fever	Dysaesthesia in arms	Stenosis and thickened walls in the subclavian, left carotid and right renal arteries	Necrotizing vasculitis with lobular panniculitis in the deep dermis
Ghosn et al. [10], Lebanon, 2008	F	Lebanese	Face, trunk and extremities	7	7	7	Solumedrol 0.5 mg/kg/d, followed by infliximab 100 mg at weeks 0, 2, and 6		Acute-onset right wrist drop	Marked aneurysmal dilatation of the ascending aorta with occlusion of the left and right subclavian arteries, in addition to a large aneurysmal pouch in the right axillary area compressing the nerve plexus	Diagnosed as RP for 5 years
Aoussar et al. [11], France, 2007	F	Caucasian	Forearm	No record	21	23	Prednisone				
Kanaemistu et al. [12], Japan, 2005	F	Japanese	Neck, chest and arm	No record	34	34	Prednisolone and cyclosporine A, surgery			Aneurysm from the ascending aorta to the aortic arch	Thoracic aortic aneurysm
Ujiie et al. [13], Japan, 2004	F	Japanese	First limited to lower legs and then extended to buttocks, lower trunk, upper limbs and scalp	21	29	21	Prednisolone 20 mg/d for TA, prednisone 50 mg/d, cyclosporine 6 mg/kg/d	Hypertension and dizziness	Diminished arterial pulse intensity and bruits	Stenosis in the right subclavian, right vertebral and right carotid arteries and occlusions in the left subclavian and left carotid arteries	
Fearfield et al. [14], U.K., 1999	F	Caucasian	Perineum, forearms and upper arms	27	27	33	Prednisone 150 mg/d, intravenous immunoglobulin, minocycline 200 mg/d, later methotrexate 7.5 mg/week and cyclosporine 5 mg/kg/d	Mild influenza- like symptoms, including general muscle aches	Heaviness in arms	Bilateral proximal subclavian artery occlusions with slight narrowing of the right vertebral artery origin	
Dagan et al. [15], Israel, 1995	M	Israeli	Face and extremities	4	9 months	4	Prednisone 2 mg/kg/d and dapsone 5 mg/kg for PG, high-dose prednisone and surgery for TA	Restless, tired and febrile	Absence of both radial pulses	Severe aneurysmatic dilation of the ascending aorta, severe stenosis of the left subclavian artery	Sterile osteomyelitis
Fullerton et al. [16], 1991	F	Saudi Arabian	Abdomen, followed by rapid spread to legs	No record	28	No record	Prednisone, cyclosporine				
Frances et al. [4], France, 1990	F	Portuguese	Left arm	32	32	33	Prednisolone 0.5 mg/kg/d	Fever, arthralgia	Absence of left radial pulse	Supra-aortic arteries, abdominal aorta	
Perniciaro et al. [17], USA, 1987	M		Left popliteal area	17.5	19	19	Systemic corticosteroids	Intermittent fever			
Case 1	F	Chinese	Bilateral thighs and buttocks, upper extremities	24	24	28	Prednisolone 40 mg/d, cyclosporine 0.1 g/d, triptolide 60 mg/d	Fever	Dizziness, pulseless in both upper limbs	The right innominate artery (subclavian artery, common carotid artery) was completely obliterated, and the left common carotid artery was entirely occluded. The initial segment of the left vertebral artery had stenosis, and the left vertebral artery was enlarged	
Case 2	F	Chinese	Both lower and upper extremities, left buccal region, face	No record	17	24	Methylprednisolone		Left arm weakness and dizziness	The left carotid artery, left subclavian artery and bilateral femoral arteries, the superficial femoral arteries and left popliteal artery, the ascending aorta,aortic arch and its branches, descending aorta, abdominal aorta. The celiac stem and mesenteric artery were occluded, with multiple collateral circulation formation. The main hepatic artery and splenic artery	

*PG* pyoderma gangrenosum, *TA* Takayasu arteritis, *RP* relapsing polychondritis

PG is a type of neutrophilic dermatosis with noninfectious ulcers characterized by neutrophil infiltration of the skin. Alghough PG may be an isolated finding, it is most often associated with ulcerative colitis, Crohn’s disease, rheumatoid arthritis, and rarely, TA [19]. Several cases have highlighted that PG is also a complication of TA [2–4, 7–18]. Of these 18 cases, the peak age of onset was between the first and third decades of life. The median age for diagnosis of pyoderma gangrenosum was 22.5 years, and that for Takayasu arteritis was 26.0 years. There is a marked female preponderance with male-female ratio of 1:3.5. PG occurred earlier than TA in 11 cases, with the median time interval of 4 years. In contrast, TA preceded PG in 3 cases, and the median time interval was also 4 years. PG and TA were diagnosed simultaneously in 3 cases, and there were no related data for one patient. Our review demonstrated that PG can occur at any stage of the disease process of TA. However, it is very difficult to establish a time relationship between the course of PG and that of TA due to the lack of specific immunological findings and the long duration of the systemic manifestations prior to the onset of vascular symptoms. The first or “prepulseless” stage of TA, characterized by nonspecific physical symptoms, arthralgia, and myalgia, was not noted at the time of PG diagnosis [20]. It may well be that the continued therapy for PG delayed the symptoms of TA. The patient in case 2 we presented with a 7-year history of PG, with irregular treatment before symptoms related to arterial occlusion appeared. This may be one reason for the severe arterial involvement. Although possible, this remarkable association and clinical development are unlikely to be coincidental, and these findings should lead physicians to consider the possible diagnosis of TA and to look for evidence of TA when diagnosing a PG patient of unknown aetiology, because early diagnosis, active treatment and regular monitoring are prudent to prevent problematic changes in multiple arteries.

Typically, PG skin lesions are more frequently observed on the lower extremities [19]. Ujiiel et al. reported that PG lesions associated with TA tend to be more widespread than are those without TA [14]. In our study, the lesions showed more extensive body involvement, including the lower extremities (72.2%), upper extremities (66.7%), and trunk, and buttock and pubic regions (50%) as well as the scalp, face and neck (50%), which is consistent with the literature [14]. There appears to be no correlation between the sites of PG and the involved large vessels shown on angiography. Therefore, the theory of arterial occlusion and skin lesions as a cause-and-effect phenomenon seems unlikely. Our data showed that the presence of skin lesions in patients with TA does not appear to be associated with a more severe disease course, which was also shown by other researchers [4].

There have been different hypotheses regarding the pathogenesis of TA, and upregulated proinflammatory cytokines, such as interleukin (IL)-6, IL-8, IL-18, and IL-23, and tumour necrosis factor-α (TNF-α), have been observed in patients with TA [21]. Interestingly, evidence of significant overexpression of IL-6, IL-8, IL-17 and TNF-α has also been found in PG lesions [5, 22]. Based on recent evidence and case studies regarding patients with TA and PG, the co-existence of these two diseases appears to be reasonable.

Our review reveled that systemic corticosteroids were used in all PG cases associated with TA. Fifteen patients were given immunosuppressive drugs to prevent disease recurrence. Systemic corticosteroids are the mainstay of treatment of patients with TA. Corticosteroids are also the primary treatment of choice for PG [13]. According to the previous literature, approximately two-thirds of cases have been successfully treated with systemic corticosteroids; the other cases were resistant to this therapy. As an alternative therapy, immunosuppressive drugs, such as azathioprine, cyclophosphamide, cyclosporine, and tacrolimus, were also demonstrated to be effective [3].

In conclusion, in addition to evidence from the available literature, our case reports illustrate the need to consider the rare possibility of underlying TA in all patients with PG, especially in young female patients. We suggest that asymptomatic young patients with such skin lesions should be examined carefully and assessed periodically, which would minimize the delay in diagnosis and initiation of therapy of TA. Early diagnosis and treatment are prudent to prevent problematic complications and improve prognosis.

## Data Availability

Data sharing is not applicable to this article because no datasets were generated or analysed during the current study.

## Abbreviations

CRP: C-reactive protein
ESR: Erythrocyte sedimentation rate
PG: Pyoderma gangrenosum
RP: Relapsing polychondritis
TA: Takayasu arteritis
